# Impacts of increasing challenge with *Eimeria maxima* on the growth performance and gene expression of biomarkers associated with intestinal integrity and nutrient transporters

**DOI:** 10.1186/s13567-021-00949-3

**Published:** 2021-06-09

**Authors:** Po-Yun Teng, Janghan Choi, Yuguo Tompkins, Hyun Lillehoj, Woo Kim

**Affiliations:** 1 Department of Poultry Science, University of Georgia, Athens, GA USA; 2 U.S. Department of Agriculture-Agricultural Research Center, Beltsville, MD USA

**Keywords:** Coccidiosis, *Eimeria maxima*, Gastrointestinal tract health, Gastrointestinal permeability, Nutrient transporters, Tight junction proteins, Broiler chickens

## Abstract

This study was conducted to investigate the impacts of graded severity of *Eimeria maxima* infection on the growth performance and intestine health of broiler chickens. Four different levels of *E. maxima*-challenged treatments were used, including a non-challenged control group, a low challenge (12 500 oocysts), a medium challenge (25 000 oocysts), and a high challenge dose (50 000 oocysts). There were eight replicate cages per treatment, with 12 birds in each cage, and chickens in the challenged groups orally received sporulated oocysts on day 14. Gastrointestinal permeability was measured by fluorescein isothiocyanate dextran at 5 days post-infection (dpi), whereas intestinal morphology and gene expression of nutrient transporters and tight junction proteins were determined at 6 dpi. The results demonstrate a linear reduction in growth performance, jejunal villus height, and jejunal integrity with graded challenge doses of *E. maxima* (*P* < 0.01). Moreover, linear regulation of nutrient transporters and tight junction proteins was a consequence of increasing *Eimeria* infection levels (*P* < 0.01). The linear increase of Claudin 1, cationic amino acid transporter, glucose transporter 1, and L-type amino acid transporter genes was associated with increased severity of coccidiosis (*P* < 0.01). Furthermore, expression of nutrient transporters located at the brush border membrane were down-regulated (*P* < 0.01) with increasing *E. maxima* inoculation dose. In conclusion, growth performance and key intestinal integrity biomarkers in broiler chickens were adversely influenced in a dose-dependent manner by *E. maxima* infection.

## Introduction

Coccidiosis, a major parasitic disease of poultry caused by several different species of the genus *Eimeria*, causing intestine damage, poor nutrient digestion, and diarrhea, costs approximately 14 billion United States dollars per year to the poultry industry [1, 2]. Among seven *Eimeria* species found in chickens, *E. maxima* mainly invade the jejunum and ileum, where most dietary nutrients are digested and absorbed. Thus, *E. maxima* infection usually shows negative impact on growth performance because of nutrient malabsorption, and in some severe cases, high mortality in commercial farms.

*Eimeria* infection is known to inhibit the activities of brush border digestive enzymes such as maltase and sucrase [2–4]. Moreover, chickens infected with *E. maxima* showed down-regulation of gene expression of nutrient transporters in the small intestine [5–7]. Nutrient transporters are protein complexes that transport dietary nutrients across the brush border membrane and the basolateral membrane of enterocytes. Glucose transporter 5 (GLUT5) is capable of transporting monosaccharides, whereas Na^+^-dependent amino acid transporter (B^0^AT), Na^+^-independent amino acid transporter (B^0+^AT), and excitatory amino acid transporter (EAAT) can transport amino acids from the intestinal lumen to intestinal epithelial cells. Simple sugars and amino acids concentrated in the enterocytes are, however, exported into blood circulation though the nutrient transporters located at the basolateral membrane, such as glucose transporter 1 (GLUT1), glucose transporter 2 (GLUT2), cationic amino acid transporter (CAT1), L-type amino acid transporter 1 (LAT1), and Na^+^-dependent neutral/cationic amino acid transporter (LAT2). A reduction in the expression of nutrient transporters and endogenous enzymes in *Eimeria*-infected chickens results in a decrease in the digestibility of amino acids, energy, fat, and minerals [8–13]. Given that *E. maxima* mainly infects the jejunum and impairs the functions of the jejunum, the main focus of this work was on evaluating *E. maxima* infection on biomarkers associated with intestinal integrity and nutrient transporters of broiler chickens.

Previous studies have demonstrated a strong relationship between the severity of *Eimeria* challenge and the growth performance of chickens [14, 15]. Additionally, digestibility of amino acids and energy was linearly decreased in a dose-dependent manner following graded *Eimeria* infection [11, 13]. However, one question that arises is what the potential impacts of increasing *E. maxima* infection levels on intestinal biomarkers are. Thus, the current study was conducted to investigate intestine health responses to four levels of *E. maxima* challenge doses. It was hypothesized that the intestinal integrity and gene expression of nutrient transporters would be linearly regulated in response to graded challenge doses of *E. maxima.* The reaction of intestinal ecosystems to different *E. maxima* infection severities would reveal possible mechanism of action by which parasites influence nutrient absorption and growth performance.

## Materials and methods

### Experimental design and sample collection

 The study was conducted at the Poultry Research Center, University of Georgia, and approved by the Institutional Animal Care and Use Committee (A2018 09-006). Five hundred male broiler chickens (Cobb 500, Cobb-Vantress, Cleveland, GA, USA) were raised and fed with corn-soybean meal-based starter diet (21% crude protein and 2,975 kcal/kg AMEn) without receiving coccidia vaccine and coccidiostats from day 0 to 14. At day 14, chickens were weighed individually. A total of three hundred and eighty-four birds that had similar body weight (420 ± 20 g) were selected before being randomly allocated to 32 cages. Four sets of battery cages were used in the study with a floor allowance of approximately 0.049 m^2^ per bird. Each cage has five tiers and three cages in a row, but the top and the bottom tiers were not used in the experiment. Broiler chickens were fed with grower diet without supplementation of coccidiostats (19% crude protein and 3,025 kcal/kg AMEn) from day 14 to day 20. Eight replicate cages were allotted for each treatment group, with 12 chickens per cage. Feed and water were provided ad libitum and experimental animals were raised in the same room where the environmental temperature was set at 27 °C on day 14 and reduced to 24 °C by decreasing 1° every other day according to the recommendation of the Cobb Broiler Management Guide [16].

There were four levels of *E. maxima*-challenged treatment groups: a low challenge dose, a medium challenge dose and a high challenge dose, and a non-challenged control group (Control). At the beginning of the study, the chickens in the low-dose treatment (Low) group were gavaged with 12 500 sporulated oocysts of *E. maxima* per bird; the chickens in the medium-dose treatment (Medium) were gavaged with 25 000 sporulated oocysts per bird; the chickens in the high-dose treatment (High) were gavaged with 50 000 sporulated oocysts per bird; whereas, the non-challenged chickens were gavaged with 1 mL of water. The dosage levels for the current study were chosen based on previous studies [11, 13].

The *E. maxima* used in the study were isolated and propagated from a North Carolina field strain. In brief, a single oocyst was obtained using a pipette under a light microscope. The chicken that was used for oocyst propagation was raised in a disinfected isolator and infected with one oocyst at the age of 14 days. At 7 dpi, feces were collected from the isolator. The oocysts were separated from feces by salt flotation and sporulated with an air pump at room temperature. Three rounds of passages were performed to gain sufficient *E. maxima* oocysts for the current experiment.

The feed intake (FI) and body weight (BW) of chickens per pen were recorded on 0 and 6-dpi to calculate body weight gain (BWG) and feed conversion rate (FCR) during the experimental period. Mortalities were recorded to adjust FI and FCR when chickens died before the trial termination. On 5 dpi, one chicken per cage was sacrificed by cervical dislocation to measure gastrointestinal permeability. On 6 dpi, three to five centimeter long sections of mid-jejunum were collected from one bird per cage. Intestinal digesta was gently flushed out of the tissue using phosphate buffer saline. Afterward, the intestinal tissue was immediately fixed in 10% formalin to determine intestinal morphology. For gene expression analysis, another five centimeters long section of mid-jejunum was collected. The jejunal mucosa was gently scraped with a blunt microslide, immediately snap-frozen in the liquid nitrogen, and stored at −80 °C until further processing.

### Gastrointestinal permeability

One chicken per cage was gavaged with 1 mL of fluorescein isothiocyanate dextran (FITC-d; 2.2 mg/mL, MW 4000; Sigma-Aldrich, Canada) to measure gastrointestinal permeability [13, 17]. At 2 h post-inoculation, blood was collected from chickens after euthanasia by cervical dislocation. Blood samples were stored in a dark container for an additional 2 h at room temperature before centrifugation at 1000 × *g* for 15 min. A hundred microliter of serum was transferred from the blood sampling tubes to a dark 96-well microplate. Five levels of FITC-d standard solution were formulated with the stock solution (2.2 mg/mL) and the pool of serum from 10 additional unchallenged chickens raised in the same house. The standard solution and test samples were measured at an excitation wavelength of 485 nm and an emission wavelength of 528 nm with a microplate reader (Spectramax M5, Molecular Devices, San Jose, CA, USA). The concentration of FITC-d in the serum was calculated using a standard curve. The processing of blood and preparation of standard solution were performed under a dark environment in order to protect FITC-d from light exposure.

### Intestinal morphology

The fixed intestinal tissues were removed from the 10% formalin and embedded in paraffin. Afterward, 4 μm tissue slides were made from the embedded samples and stained with hematoxylin and eosin. The intestinal morphology of each slide was observed and captured by a light microscope with 2× magnification (BZ-X800, Keyence Inc, Itasca, IL, USA). Villus height and crypt depth was measured by Image J (Image Processing and Analysis in Java–ImageJ 1.50i, National Institutes of Health) with five representative villi and crypts per slide. Additionally, the ratio of villi height to crypt depth was calculated by the average value of villi height and crypt depth from each slide.

### Real-time PCR analysis

Total RNA from each sample was extracted and homogenized in QIAzol Lysis Reagents (Qiagen, Germantown, MD, USA) with a bead beater. The RNA quantity was determined by a NanoDrop 2000 spectrophotometer (Thermo Fisher Scientific, MA, USA). The extracted RNA was diluted to the same concentration for each sample and reverse-transcribed to cDNA by the High Capacity cDNA synthesis kits (Applied BioSystems, Life Technologies, CA, USA). In the real-time PCR reactions, cDNA samples were mixed with SYBR Green Master mix (Bio-Rad Laboratories, Hercules, CA, USA) for analysis in the Step One thermo-cycler (Applied Biosystem, Foster City, CA, USA) in duplicates. The 2^−ΔΔCt^ method was used to analyze target gene expression compared to a housekeeping gene [18]. GAPDH was selected to be the reference gene based on the most stable Ct value among other housekeeping genes that has been tested (beta-actin, 18S, and hydroxymethylbilane synthase). The variation of Ct value was within one Ct-cycle among all treatments and replicates. Additionally, there was no linear effect observed in the Ct value of the reference gene in response to the graded levels of *Eimeria* challenge doses. The mean ΔCt from the non-challenged control was used as the base standard to adjust the fold changes of other treatments. The integral proteins and membrane associated guanylate kinase homologue proteins (ZO family) at the tight junction complex were evaluated in the study, including occludin (OCLDN), Zonula occludens 1 (ZO1), Zonula occludens 2 (ZO2), junctional adhesion molecule 2 (JAM2), claudin 1 (CLDN1), and claudin 2 (CLDN2). Moreover, gene expression of mucin (mucin 2, MUC2), monosaccharide transporters (GLUT1, GLUT2, GLUT5, and, SGLT1), and amino acids transporters (B^0^AT, B^0+^AT, EAAT, CAT1, LAT1, and LAT2) were determined in the mucosa of the jejunum. The sequences of the forward and reverse primers are listed in Table 1.

**Table 1:** **List of primers used for qPCR**

Gene symbol	Accession Number	Forward primer	Reverse primer
GAPDH^a^	NM_204305.1	CCTCTCTGGCAAAGTCCAAG	GGTCACGCTCCTGGAAGATA
CLDN1^b^	NM_001013611.2	TGGAGGATGACCAGGTGAAGA	CGAGCCACTCTGTTGCCATA
CLDN2^b^	NM_001277622.1	CCTGCTCACCCTCATTGGAG	GCTGAACTCACTCTTGGGCT
OCLDN^b^	NM_205128.1	ACGGCAGCACCTACCTCAA	GGCGAAGAAGCAGATGAG
ZO1^b^	XM_015278981.2	CAACTGGTGTGGGTTTCTGAA	TCACTACCAGGAGCTGAGAGGTAA
ZO2^b^	NM_204918.1	ATCCAAGAAGGCACCTCAGC	CATCCTCCCGAACAATGC
JAM2^b^	NM_001006257.1	AGCCTCAAATGGGATTGGATT	CATCAACTTGCATTCGCTTCA
MUC2^c^	JX284122.1	ATGCGATGTTAACACAGGACTC	GTGGAGCACAGCAGACTTTG
GLUT5 (SLC2A5)^d^	XM_417596.6	TTGCTGGCTTTGGGTTGTG	GGAGGTTGAGGGCCAAAGTC
SGLT1 (SLC5A1)^d^	NM_001293240.1	GCCGTGGCCAGGGCTTA	CAATAACCTGATCTGTGCACCAGT
B^0^AT (SLC6A19)^d^	XM_419056.6	GGGTTTTGTGTTGGCTTAGGAA	TCCATGGCTCTGGCAGAGAT
B^0+^AT (SLC7A9)^d^	NM_001199133.1	CAGTAGTGAATTCTCTGAGTGTGAAGCT	GCAATGATTGCCACAACTACCA
EAAT (SLC1A1)^d^	XM_424930.6	TGCTGCTTTGGATTCCAGTGT	AGCAATGACTGTAGTGCAGAAGTAATATATG
GLUT2 (SLC2A2)^d^	NM_207178.1	TCATTGTAGCTGAGCTGTT	CGAAGACAACGAACACATAC
CAT1 (SLC7A1)^d^	XM_015277945.2	CCAAGCACGCTGATAAAG	TACTCACAATAGGAAGAAGGG
LAT2 (SLC7A6)^d^	XM_025154295.1	TCAGCTTCAGTTACTGGTT	GCACAACCACGAGAAATAC
GLUT1(SLC2A1)^d^	NM_205209.1	CTTTGTCAACCGCTTTGG	TGTGCCCCGGAGCTTCT
LAT1 (SLC7A5)^d^	NM_001030579.2	GATTGCAACGGGTGATGTGA	CCCCACACCCACTTTTGTTT

GAPDH glyceraldehyde-3-phosphate dehydrogenase, CLDN1 Claudin 1 [49], CLDN2 Claudin 2 [50], OCLDN Occludin [51], ZO1 Zonula occludens 1 [52], ZO2 Zonula occludens 2 [53], JAM2 Junctional adhesion molecule 2 [48], MUC2 Mucin [47], GLUT5 Glucose transporter 5, SGLT1 sodium glucose transporter 1*,* B^0^AT Na^+^-dependent amino acid transporter, B^0+^AT Na^+^-independent amino acid transporter, EAAT Excitatory amino acid transporter, GLUT2 Glucose transporter 2, CAT1 Cationic amino acid transporter, LAT2 Na^+^-dependent neutral/cationic amino acid transporter*,* GLUT1 Glucose transporter 1, LAT1 L-type amino acid transporter 1*.*

^a^Housekeeping gene.

^b^Tight junction proteins.

^c^Mucin.

^d^Nutrient transporters [24].

### Statistical analyses

All data were analyzed in the PROC GLM program of SAS software (SAS Institute Inc., Cary, NC, USA). For bird mortality, the number of dead birds per pen was transformed by the formula below [19].

$${\text{Transformed}}\,{\text{data}} = \sqrt {{\text{Dead birds}} + 1}$$

Orthogonal polynomial contrasts were used to evaluate the linear and quadratic impacts of graded *E. maxima* challenge doses on growth performance, mortality, and parameters of intestine health. The CONTRAST statements within PROC GLM were used to perform the analyses and the statistical significance was set at *P* < 0.05 [20].

## Results

### Growth performance and mortality

Increasing the inoculation doses of *E. maxima* linearly and quadratically decreased the BW, BWG, and FI of birds (*P* < 0.0001, Table 2). Additionally, the graded challenge levels resulted in linear increases in FCR from 0 to 6 dpi (*P* < 0.0001). The FCR for the High group increased from 1.40 to 3.14 compared to the Control group. The chickens challenged with *E. maxima* had a 62% reduction in BWG compared to the Control group. Moreover, the mortality of birds was linearly increased in a dose-dependent manner (*P* < 0.0001).

**Table 2:** **Effects of graded E. maxima infection on growth performance and mortality of broiler chickens from 0 to 6-dpi**

Items^a^	Control	Low	Medium	High	Linear^b^*P* value	Quadratic^b^ *P* value
BW (g)	832 ± 8^c^	637 ± 5	565 ± 10	524 ± 6	< 0.0001	< 0.0001
BWG (g)	415 ± 8	203 ± 5	139 ± 9	132 ± 6	< 0.0001	< 0.0001
FI (g)	579 ± 9	435 ± 17	414 ± 17	410 ± 16	< 0.0001	< 0.0001
FCR	1.40 ± 0.02	2.15 ± 0.07	3.04 ± 0.15	3.14 ± 0.12	< 0.0001	< 0.0001
Mortality (%)	1.04 ± 1.04	4.17 ± 1.57	19.79 ± 1.04	23.96 ± 1.04	< 0.0001	0.0713

The study evaluated the effects of graded challenge of *E. maxima* on growth performance and gastrointestinal biomarkers in the broiler chickens. Birds were challenged with *E. maxima* on day 14.

^a^Low, 12 500 oocysts of *E. maxima*; Medium, 25 000 oocysts of *E. maxima*; High, 50 000 oocysts of *E. maxima*; BW body weight, BWG body weight gain, FI feed intake, FCR feed conversion rate.

^b^The *P* values indicate whether the analyzed parameters linearly or quadratically responded to the graded challenge doses of *E. maxima*.

^c^Mean ± SD.

### Intestinal morphology

**Figure 1:**
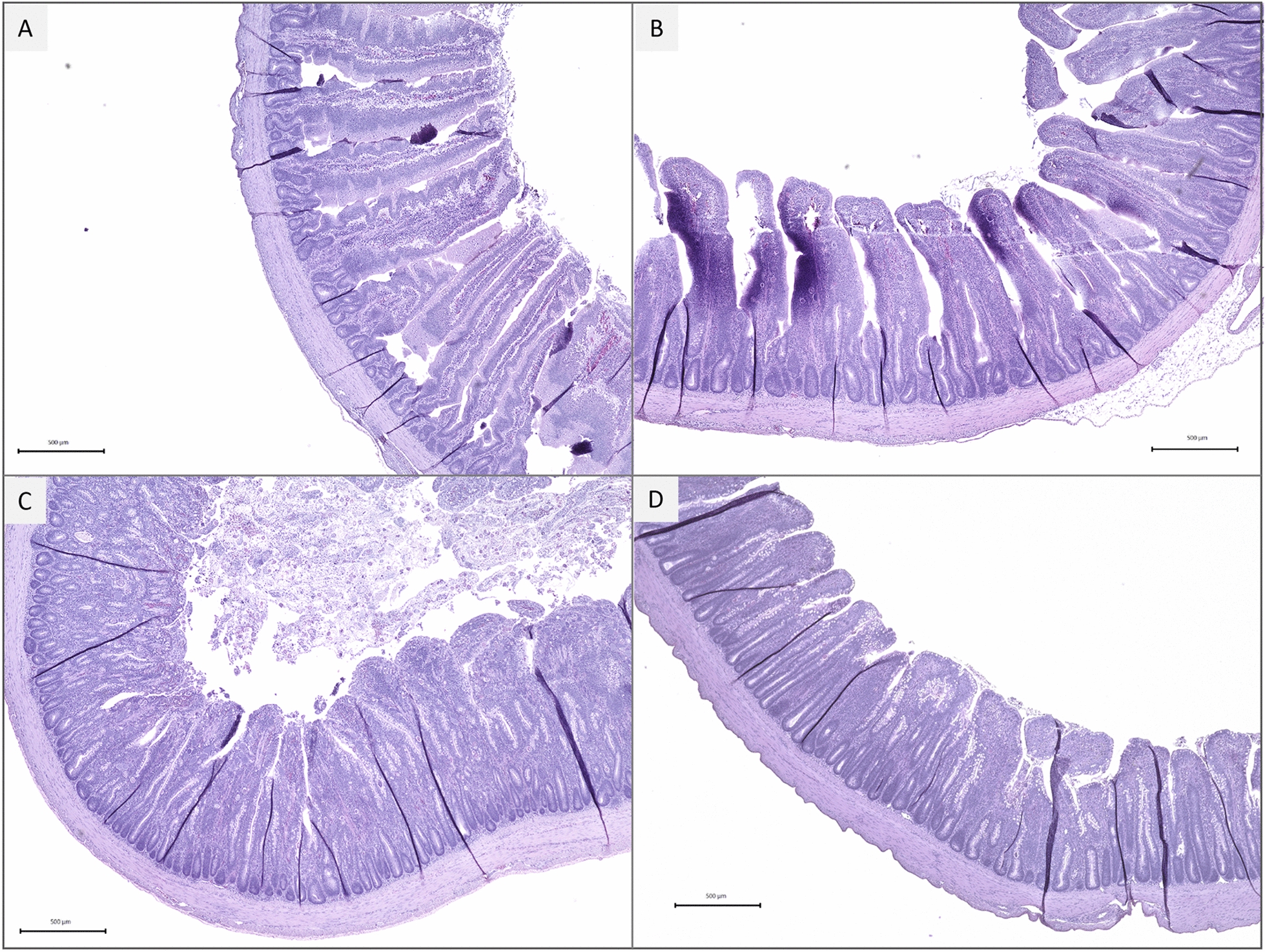
**Effects of graded *E. maxima* infection on intestinal morphology (jejunum) of broiler chickens at 6-dpi. A** Control; **B** Low; **C** Medium; **D** High. The study evaluated the effects of graded challenge of *E. maxima* on growth performance and gastrointestinal biomarkers in the broiler chicken. Birds were challenged with *E. maxima* on day 14. Low, 12 500 oocysts of *E. maxima*; Medium, 25 000 oocysts of *E. maxima*; High, 50 000 oocysts of *E. maxima*.

Linear reductions for villus height in the jejunum were observed with increasing challenge oocyst doses (*P* < 0.0001; Table 3, Figure 1). In addition, the crypt depth of the jejunum also exhibited a linear response to the graded *E. maxima* infection (*P* < 0.05), whereas the ratio of villus height to crypt depth in the jejunum showed a trend of a quadratic effect (*P* < 0.1). Furthermore, the chickens challenged with Low or High doses of *E. maxima* exhibited numerically lower ratio of villi height to crypt depth in the jejunum compared to the Control group.

**Table 3:** **Effects of graded E. maxima infection on intestinal morphology of broiler chickens on 6-dpi**

Items^a^	Control	Low	Medium	High	Linear^b^ *P* value	Quadratic^b^ *P* value
*Jejunum* (μm)						
Villi height	1155 ± 46^d^	911 ± 27	900 ± 31	800 ± 22	< 0.0001	0.006
Crypt depth	205 ± 9	216 ± 34	182 ± 6	171 ± 7	0.002	0.804
Ratio^c^	5.85 ± 0.35	4.48 ± 0.26	5.15 ± 0.22	4.96 ± 0.20	0.143	0.060

The study evaluated the effects of graded challenge of *E. maxima* on growth performance and gastrointestinal biomarkers in the broiler chickens. Birds were challenged with *E. maxima* on day 14. Intestinal tissue was collected on 6-dpi.

^a^Low, 12 500 oocysts of *E. maxima*; Medium, 25 000 oocysts of *E. maxima*; High, 50 000 oocysts of *E. maxima*.

^b^The *P* values indicate whether the analyzed parameters linearly or quadratically responded to the graded challenge doses of *E. maxima*.

^c^Ratio of villi height to crypt depth

^d^Mean ± SD.

### Gastrointestinal permeability and gene expression of tight junction proteins

**Figure 2:**
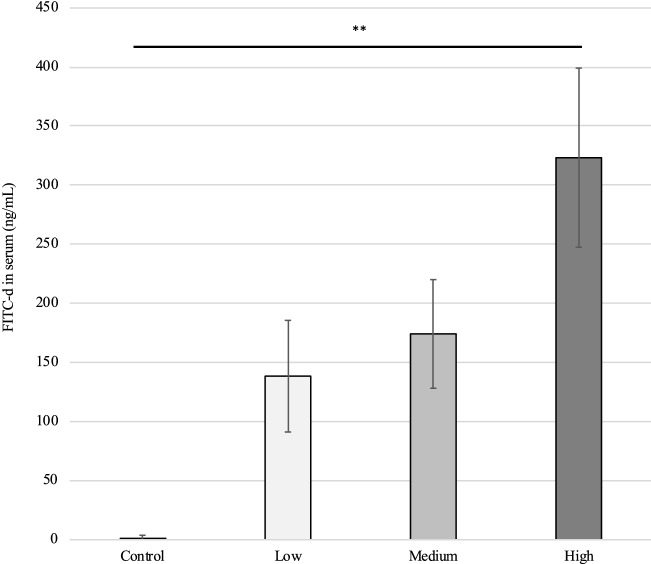
**Effects of graded *E. maxima* infection on intestinal permeability of broiler chickens on 5-dpi measured by fluorescein isothiocyanate dextran.** **Significant linear effects, *P* < 0.01. *N* = 8. The study evaluated the effects of graded challenge of *E. maxima* on growth performance and gastrointestinal biomarkers in broiler chickens. Birds were challenged with *E. maxima* on day 14. Low, 12 500 oocysts of *E. maxima*; Medium, 25 000 oocysts of *E. maxima*; High, 50 000 oocysts of *E. maxima.* The error bars represent the SD values.

**Figure 3:**
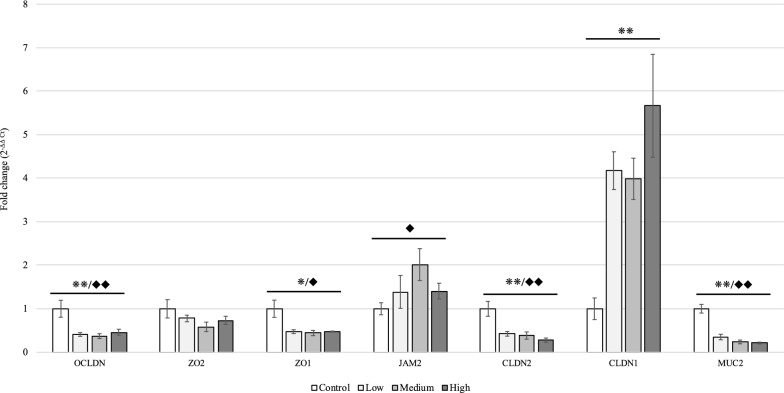
**Effects of graded *E. maxima* infection on gene expression of tight junctions and mucin in the mucosa of jejunum in broiler chickens on 6-dpi.** */**Significant linear effects, *P < 0.05, **P < 0.01. ^◆^/^◆◆^Significant quadratic effects, ^◆^*P* < 0.05, ^◆◆^*P* < 0.01. *N* = 8. The study evaluated the effects of graded challenge of *E. maxima* on growth performance and gastrointestinal biomarkers in broiler chickens. Birds were challenged with *E. maxima* on day 14. Low, 12 500 oocysts of *E. maxima*; Medium, 25 000 oocysts of *E. maxima*; High, 50 000 oocysts of *E. maxima*; OCLDN, occludin; ZO2, Zonula occludens 2; ZO1, Zonula occludens 1; JAM2, junctional adhesion molecule 2; CLDN2, claudin 1; CLDN1, claudin 1; MUC2, intestinal mucin 2*.* The error bars represent the SD values.

The results of gastrointestinal permeability at 5 dpi) (Figure 2) indicated that increasing the severity of *E. maxima* infection linearly elevated the FITC-d levels in the serum (*P* < 0.01), suggesting that gastrointestinal leakage was linearly increased in response to higher levels of *E. maxima* challenge. Furthermore, linear responses were observed for the gene expression of several tight junction proteins and the mucin at 6 dpi) (Figure 3). The mRNA levels of OCLDN, ZO1, CLDN2, and MUC2 were linearly and quadratically decreased, whereas the gene expression of CLDN1 was significantly upregulated when birds were challenged with higher levels of oocysts (*P* < 0.01). In addition, there was a quadratic effect on the gene expression of JAM2 in the mucosa of the jejunum (*P* < 0.05). JAM2 was upregulated in the Medium group at levels two times higher than that in the Control group. However, there was no significant difference in gene expression of ZO2 among all treatments.

### Gene expression of nutrient transporters

**Figure 4:**
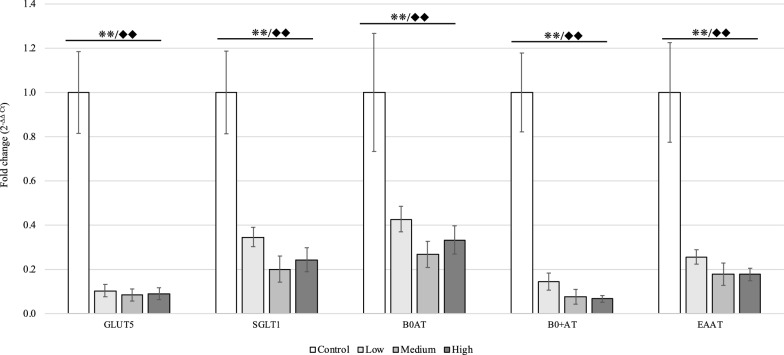
**Effects of graded *E. maxima* infection on gene expression of nutrient transporters (brush border) in the mucosa of jejunum in broiler chickens on 6-dpi.** */**Significant linear effects, *P < 0.05, **P < 0.01. ^◆^/^◆◆^Significant quadratic effects, ^◆^*P* < 0.05, ^◆◆^*P* < 0.01. *N* = 8. The study evaluated the effects of graded challenge of *E. maxima* on growth performance and gastrointestinal biomarkers in the broiler chickens. Birds were challenged with *E. maxima* on day 14. Low, 12 500 oocysts of *E. maxima*; Medium, 25 000 oocysts of *E. maxima*; High, 50 000 oocysts of *E. maxima*; GLUT5, glucose transporter 5; SGLT1, sodium glucose transporter1; B^0^AT, Na^+^-dependent amino acid transporter; B^0+^AT, Na^+^-independent amino acid transporter; EAAT, Excitatory amino acid transporter. The error bars represent the SD values.

**Figure 5:**
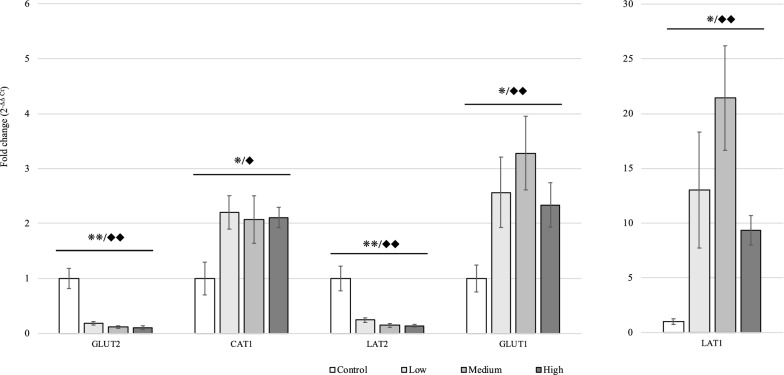
**Effects of graded *E. maxima* infection on gene expression of nutrient transporters (basolateral membrane) in the mucosa of jejunum in broiler chickens on 6-dpi.** */**Significant linear effects, *P < 0.05, **P < 0.01. ^◆^/^◆◆^Significant quadratic effects, ^◆^*P* < 0.05, ^◆◆^*P* < 0.01. *N* = 8. The study evaluated the effects of graded challenge of *E. maxima* on growth performance and gastrointestinal biomarkers in the broiler chickens. Birds were challenged with *E. maxima* on day 14. Low, 12 500 oocysts of *E. maxima*; Medium, 25 000 oocysts of *E. maxima*; High, 50 000 oocysts of *E. maxima;* GLUT2, glucose transporter 2; GLUT1, glucose transporter 1; CAT1, Cationic amino acid transporter; LAT2, Na^+^-dependent neutral/cationic amino acid transporter; LAT1, L-type amino acid transporter 1. The error bars represent the SD values.

Gene expression of nutrient transporters located at the brush border of the mucosa was linearly and quadratically reduced in response to increasing doses of *Eimeria* challenge (*P* < 0.01) (Figure 4). The challenged chickens exhibited reduced gene expression for GLUT5 (Low, 90% of expression reduction compared to the non-challenged group; Medium, 92%; High, 91%), SGLT1(Low, 75% of expression reduction compared to the non-challenged group; Medium, 80%; High, 76%), B^0^AT (Low, 57% of expression reduction compared to the non-challenged group; Medium, 73%; High, 66%), B^0+^AT (Low, 86% of expression reduction compared to the non-challenged group; Medium, 92%; High, 93%), and EAAT (Low, 74% of expression reduction compared to the non-challenged group; Medium, 82%; High, 82%) compared to the Control group. Moreover, linear and quadratic reductions in GLUT2 and LAT2 was observed in the basolateral membrane of mucosa in response to the graded increasing level of *E. maxima* infection (*P* < 0.01). Gene expression of GLUT2 and LAT2 in challenged chickens was downregulated to less than 20% of the Control group. CAT1, GLUT1, and LAT1, located at the basolateral membrane, were, however, linearly and quadratically upregulated with increasing inoculation doses of *E. maxima* (*P* < 0.05, Figure 5)*.* The gene expression of LAT1 in the Medium group was 21 times higher than that in the Control group, whereas those of CAT1 and GLUT1 were increased to 207% and 328%, respectively, in the Control group (Figure 5).

## Discussion

Linear reduction in growth performance in response to the increase in inoculation doses of *E. maxima* could be attributed to several factors. First, FI plays a crucial role in maintaining the intestinal health status and BWG of chickens. In the present study, FI was significantly decreased in the challenged chickens because of sickness and lethargy after the *E. maxima* infection. The linear decrease in feed intake was strongly associated with BW when chickens were challenged with graded levels of mixed *Eimeria* spp. [13]. It was reported that the correlation coefficient between BW and FI was up to 0.8 in our previous study [13]. Moreover, a meta-analysis indicated that reduced FI resulted in approximately 90% of the loss of total BWG in chickens infected by *Eimeria* spp. [21]. Apart from *E. maxima* infection, increasing challenge levels of *E. acervulina*, *E. tenella*, or mixed *Eimeria* species all caused linear inhibition of the growth performance of chickens [11, 14]. However, among seven species, *E. maxima* had the most significant influence on BWG because of its preferable infection sites at the jejunum and ileum, where most dietary nutrients are supposed to be digested and absorbed [21].

The reduced nutrient digestibility could be another factor that contributes to the poor growth performance in chickens challenged with coccidia. It has been reported that *Eimeria* infection inhibits the apparent ileal digestibility of amino acids, energy, and minerals [11–13]. The decrease in nutrient digestibility in coccidiosis might be associated with the expression of nutrient transporters in the jejunum. The present results demonstrate that the increasing severity of *E. maxima* infection linearly reduced the expression of nutrient transporters located at the brush border of epithelial cells. The downregulation of GLUT5 and SGLT1 could decrease fructose, glucose, and galactose absorption [22]. Additionally, the increase in *E. maxima* challenge linearly reduced the gene expression of B^0^AT, B^0+^AT, and EAAT. B^0^AT is a nutrient transporter that takes up a broad range of neutral amino acids from the intestinal lumen to epithelial cells; thus, a reduced expression of B^0^AT decreases the transport of neutral amino acids [23, 24]. Moreover, the reduced levels of neutral amino acids further influence the uptake of cationic amino acids because neutral amino acids accumulate in enterocytes to exchange cationic amino acids by the amino acid exchanger complex composed of B^0+^AT and rBAT [23]. The downregulation of B^0+^AT in the current study indicates a depressed uptake of essential cationic amino acids, such as Arg and Lys, in response to *E. maxima* infection. These amino acids are important for the growth of the host and the development of immunity and antioxidative responses; therefore, the decreased availability of cationic amino acids might further impact host health status, such as immune responses against pathogen invasion and the growth performance of chickens [25–27]. Gene expression of EAAT, a sodium-dependent nutrient transporter for anionic amino acids including glutamate and aspartic acid [28], was also linearly reduced in response to the increase in challenge doses in the present study. It is speculated that the downregulation of EAAT reduced the uptake of glutamate which would accelerate cell death and inhibit parasite replication [6] because glutamate is the main source of energy metabolism in enterocytes. In the present study, although the expression of nutrient transporters located at the brush border was linearly reduced with increasing levels of challenge, it should be noted that the chickens in the Low group decreased the expression of the target genes to less than 50% of the Control group. The results suggest that coccidiosis causes significant impacts on the gene expression of nutrient transporters regardless of the severity of the infection.

Similar to the expression of nutrient transporters located at the brush border of the enterocytes, GLUT2, CAT1, LAT1, GLUT1, and LAT2 at the basolateral membrane were linearly regulated by increasing *E. maxima* infection in the current study. The gene expression of GLUT2 and LAT2 was downregulated, but CAT1, GLUT1, and LAT1 were upregulated by coccidiosis. The current results were in agreement with a previous study that reported increased expression of GLUT1, LAT1, and CAT1 in the jejunum after chickens were challenged with *E. maxima* [6]. Furthermore, it was reported that the expression of LAT2 and GLUT2 is reduced in the jejunum of laying hens by coccidia infection [5]. GLUT1 and GLUT2 are capable of transporting hydrolyzed carbohydrate molecules from enterocytes to the blood circulation, whereas LAT1, LAT2, and CAT transport amino acids. The mechanism of action by which coccidiosis increased GLUT1, LAT1, and CAT1, but decreased GLUT2 and LAT2 remains, however, unknown.

In the current study, the gene expression of LAT1 was increased 13-, 21-, and ninefold in the Low, Medium, and High groups, respectively. Similarly, it was reported that *E. maxima* infection causes a 19-fold change in LAT1 compared to that in non-challenged chickens [7]. LAT1 transports neutral, branched, and aromatic amino acids, such as Met and Leu. The upregulated expression of LAT1 enhances the efflux of neutral amino acids from epithelial cells. Moreover, the expression of CAT1, which transports cationic amino acids such as Cys, Arg, and Lys, was linearly increased in response to graded *E. maxima* challenge. The current findings were similar to those of studies indicating the upregulation of CAT1 and LAT1 after *Eimeria* infection [24, 29]. Both studies suggested that coccidiosis may deplete essential amino acids in infected cells by upregulating amino acid transporters at the basolateral membrane and downregulating nutrient transporters at the brush border membrane. Nutrient depletion, such as glutamine deficiency, may trigger spontaneous apoptosis in intestinal epithelial cell lines [30, 31]. The “apoptosis-induced proliferation” is crucial for tissue regeneration and recovery [32]. It has been reported that coccidiosis could accelerate the renewal of intestinal epithelial cells [33, 34]. Moreover, the increased epithelial cell turnover rate may expel parasites from the host’s intestine [35]; therefore, regulating gene expression in the enterocytes may lead to nutrient depletion and cell apoptosis which remove parasites from the host with the assistance of an increased proliferation of epithelial cells. Furthermore, Su et al. [5] have proposed two possible mechanisms that would cause the alternations of nutrient transporters during coccidiosis. They speculated that *Eimeria* infection could directly influence gene expression of epithelial cells; otherwise, the regulations could be cell-mediated consequences in response to parasite invasion. None of the hypotheses has been proven, but the latter possibility is favored by the authors.

Even though significant linear effects were observed for CAT1 and LAT1, the Medium group had numerically greater gene expression than the High group in the present study. These findings might be attributed to the crowding effect when there was no available room in the intestine of the host for parasites to develop their life cycles [36]. The numbers of parasites in the high challenge treatment may exceed the threshold of the host’s capability that could be utilized for *Eimeria*’s replication. The crowding effect might not only influence the expression of CAT1 and LAT1 in the High challenge treatment, but also the SGLT1, B0AT, and EAAT, as these target genes did not perform exact linear trends according to the increasing infection severity. Our previous study also observed that the crowding effect would influence intestinal permeability. Severe *Eimeria* infection would lead to an early peak of gastrointestinal leakage at 5 dpi, instead of 6 dpi, whereas mild infection reached the peak a day later [13]. The regulation of nutrient transporters by a crowding effect may further influence nutrient digestibility of chickens. The birds that received a medium inoculation dose of *Eimeria* had lower digestibility of amino acids and energy than those challenged with a relative higher dose [11, 13]. Because the intestinal ecosystems changed dramatically during *Eimeria* infection, further research might be needed to determine the daily expression of nutrient transporters and nutrient digestibility to understand the dynamic change of intestine status in response to different *Eimeria* infection levels. Although the threshold of parasite development still remains unclear, the current study could conclude that the transportation of amino acids and carbohydrates across the brush border and basolateral membrane was linearly responding to the graded challenge doses of *E. maxima*, but the linear trend might be influenced by a crowding effect, especially at the high challenge dose.

Coccidiosis has shown significant impacts on the intestinal integrity of chickens. The graded oocyst inoculation caused cell destruction in a dose-dependent manner, consequently influencing the gene expression of tight junction proteins and gastrointestinal permeability. FITC-d was used to determine the intestinal integrity of chickens in the current study. The greater amounts of FITC-d in the serum indicates more severe intestine damage caused by *Eimeria* infection. Our findings suggest that gastrointestinal permeability was linearly enhanced in response to graded *E. maxima* challenge. The current results agreed with previous studies that reported the linear regulation of gastrointestinal permeability with increasing *Eimeria* infection [11, 13].

Coccidiosis influences transcellular translocation by impairing intestinal epithelial cells as well as paracellular translocation by breaking the tight junctions between enterocytes. Tight junction proteins construct a complex structure connecting cells to impede the transportation of large molecules across the intestinal border. This complex is composed of several types of proteins, such as CLDN, OCLDN, JAM, and ZO families [37]. The current findings show that the expression of all tight junction proteins except ZO2 was significantly changed during coccidiosis. CLDN1 was linearly and JAM2 was quadratically increased at 6 dpi in response to graded *E. maxima* challenge. The CLDN and JAM families are responsible for tight junction strand formation and membrane apposition, respectively [38]. Moreover, both CLDN and JAM families coordinately regulate tight junction complex formation and paracellular translocation [38]. The activation of CLDN1 might be associated with the increase in cytokines caused by parasitic infection. TNF-alpha and IFN-gamma have been concluded to be the main cytokines produced by the host against *Eimeria* spp. [39, 40]. It was reported that TNF-alpha and IFN-gamma enhance the expression of CLDN1 [41, 42], and decrease OCLDN and ZO1 during inflammatory intestinal disease [43]. The results of the present study were in agreement with these previous findings demonstrating that the graded *E. maxima* infection linearly increased gene expression of JAM2 and CLDN1 but decreased ZO1, OCLDN, and CLDN2. The ZO family is a crucial adapter that connects the other tight junction proteins with cytoskeleton, constructing the intracellular domains of the protein complex [44]. Several binding partners exist for ZO-1, including CLDN, OCLDN, cingulin, and JAM1 [45]. Similarly, ZO-2 co-immunoprecipitates with ZO-1 also presenting binding sections with CLDN and OCLDN [45]. A recent in vitro study revealed that ZO1 is dominant in the tight junction protein complex in the Madin-Darby bovine kidney cells [46]. It is speculated that ZO-1 may be superior among the ZO family in the tight junction complex of chicken enterocytes, hereby, in the current study, ZO-1 was linearly reduced in response to coccidiosis, whereas ZO-2 was numerically decreased without obtaining significant difference. In addition to the tight junction proteins, the gene expression of MUC2 was linearly reduced following the increased levels of *Eimeria* inoculation in the current study. A previous study speculated that decreasing mucin production would prevent further intestinal deterioration by limiting *Clostridium perfringens* infection, as the pathogen tends to utilize mucin for replication [47]. These findings were consistent with another previous report, suggesting that the gene expression of MUC2 is reduced as a consequence of intestinal necrosis [48].

A significant linear reduction in villus height in the jejunum was observed in response to the graded doses of *E. maxima* in the present study*.* Because of the reduced absorptive surface area of the brush border in the small intestine, *Eimeria*-infected chickens possess less space to absorb nutrients from the intestinal lumen. In addition to the short villi in the jejunum, downregulation of nutrient transporters and enzymes at the brush border membrane also contribute to the reduced digestibility of amino acids and energy in coccidiosis [6, 11, 12]. Our previous research also demonstrated a strong correlation (r = 0.76) between BW and the ratio of villus height to crypt depth in *Eimeria*-infected chickens [13], suggesting that intestinal morphology is associated with chicken nutrient digestibility and growth performance.

In conclusion, graded levels of *E. maxima* infection linearly reduced growth performance and downregulated the expression of nutrient transporters located at the brush border. However, the gene expression of LAT1, CAT1, and GLUT1 was upregulated with increasing challenge doses. Linear regulation of tight junction proteins was observed in the current study. This finding was also associated with a linear increase in gastrointestinal permeability in response to the severity of *Eimeria* infection. Moreover, the greater the number of oocysts challenged to chickens was, the lower the villus height in the jejunum was. Overall, the present study demonstrates the significant impact of graded severity of coccidiosis on several intestine health biomarkers, suggesting that overall reduction of growth performance would be the combined outcome of the reduction of feed intake, gene expression of nutrient transporters, and tight junction proteins by *E. maxima* infection. The current findings would also help with deciding on an appropriate *E. maxima* challenge dose in future studies on the evaluation of feed ingredients or feed additives on improving the intestinal health of modern broiler chickens.

## Abbreviations

Control: Non-challenged control group
High: High-dose treatment
Medium: Medium-dose treatment
Low: Low-dose treatment
FI: Feed intake
BW: Body weight
dpi: Days post-infection
BWG: Body weight gain
FCR: Feed conversion rate
FITC-d: Fluorescein isothiocyanate dextran
OCLDN: Occludin
ZO1: Zonula occludens 1
ZO2: Zonula occludens 2
JAM2: Junctional adhesion molecule 2
CLDN1: Claudin 1
CLDN2: Claudin 2
MUC2: Mucin 2
GLUT5: Glucose transporter 5
SGLT1: Sodium glucose transporter 1
B^0^AT: Na^+^-dependent amino acid transporter
B^0^^+^AT: Na^+^-independent amino acid transporter
EAAT: Excitatory amino acid transporter
GLUT1: Glucose transporter 1
GLUT2: Glucose transporter 2
CAT1: Cationic amino acid transporter
LAT2: Na^+^-dependent neutral/cationic amino acid transporter
LAT1: L-type amino acid transporter 1
